# Real-world validation of smartphone-based photoplethysmography for rate and rhythm monitoring in atrial fibrillation

**DOI:** 10.1093/europace/euae065

**Published:** 2024-04-17

**Authors:** Henri Gruwez, Daniel Ezzat, Tim Van Puyvelde, Sebastiaan Dhont, Evelyne Meekers, Liesbeth Bruckers, Femke Wouters, Michiel Kellens, Hugo Van Herendael, Maximo Rivero-Ayerza, Dieter Nuyens, Peter Haemers, Laurent Pison

**Affiliations:** Department of Cardiology, Ziekenhuis Oost-Limburg, Synaps Park 1, 3600 Genk, Belgium; Department of Cardiovascular Sciences, KU Leuven, Leuven, Belgium; Limburg Clinical Research Center, Faculty of Medicine and Life Sciences, Hasselt University, Martelarenlaan 42, 3500 Hasselt, Belgium; Limburg Clinical Research Center, Faculty of Medicine and Life Sciences, Hasselt University, Martelarenlaan 42, 3500 Hasselt, Belgium; Department of Cardiovascular Sciences, KU Leuven, Leuven, Belgium; Department of Cardiology, Ziekenhuis Oost-Limburg, Synaps Park 1, 3600 Genk, Belgium; Limburg Clinical Research Center, Faculty of Medicine and Life Sciences, Hasselt University, Martelarenlaan 42, 3500 Hasselt, Belgium; Department of Cardiology, Ziekenhuis Oost-Limburg, Synaps Park 1, 3600 Genk, Belgium; Department of Cardiovascular Sciences, KU Leuven, Leuven, Belgium; Limburg Clinical Research Center, Faculty of Medicine and Life Sciences, Hasselt University, Martelarenlaan 42, 3500 Hasselt, Belgium; Research Institute Center for Statistics (CENSTAT), Hasselt University, Hasselt, Belgium; Limburg Clinical Research Center, Faculty of Medicine and Life Sciences, Hasselt University, Martelarenlaan 42, 3500 Hasselt, Belgium; Limburg Clinical Research Center, Faculty of Medicine and Life Sciences, Hasselt University, Martelarenlaan 42, 3500 Hasselt, Belgium; Department of Cardiology, Ziekenhuis Oost-Limburg, Synaps Park 1, 3600 Genk, Belgium; Department of Cardiology, Ziekenhuis Oost-Limburg, Synaps Park 1, 3600 Genk, Belgium; Department of Cardiology, Ziekenhuis Oost-Limburg, Synaps Park 1, 3600 Genk, Belgium; Department of Cardiovascular Sciences, KU Leuven, Leuven, Belgium; Department of Cardiology, Ziekenhuis Oost-Limburg, Synaps Park 1, 3600 Genk, Belgium; Limburg Clinical Research Center, Faculty of Medicine and Life Sciences, Hasselt University, Martelarenlaan 42, 3500 Hasselt, Belgium

**Keywords:** Atrial fibrillation, Mobile health, Photoplethysmography, Electrocardiography, Smartphone

## Abstract

**Aims:**

Photoplethysmography- (PPG) based smartphone applications facilitate heart rate and rhythm monitoring in patients with paroxysmal and persistent atrial fibrillation (AF). Despite an endorsement from the European Heart Rhythm Association, validation studies in this setting are lacking. Therefore, we evaluated the accuracy of PPG-derived heart rate and rhythm classification in subjects with an established diagnosis of AF in unsupervised real-world conditions.

**Methods and results:**

Fifty consecutive patients were enrolled, 4 weeks before undergoing AF ablation. Patients used a handheld single-lead electrocardiography (ECG) device and a fingertip PPG smartphone application to record 3907 heart rhythm measurements twice daily during 8 weeks. The ECG was performed immediately before and after each PPG recording and was given a diagnosis by the majority of three blinded cardiologists. A consistent ECG diagnosis was exhibited along with PPG data of sufficient quality in 3407 measurements. A single measurement exhibited good quality more often with ECG (93.2%) compared to PPG (89.5%; *P* < 0.001). However, PPG signal quality improved to 96.6% with repeated measurements. Photoplethysmography-based detection of AF demonstrated excellent sensitivity [98.3%; confidence interval (CI): 96.7–99.9%], specificity (99.9%; CI: 99.8–100.0%), positive predictive value (99.6%; CI: 99.1–100.0%), and negative predictive value (99.6%; CI: 99.0–100.0%). Photoplethysmography underestimated the heart rate in AF with 6.6 b.p.m. (95% CI: 5.8 b.p.m. to 7.4 b.p.m.). Bland–Altman analysis revealed increased underestimation in high heart rates. The root mean square error was 11.8 b.p.m.

**Conclusion:**

Smartphone applications using PPG can be used to monitor patients with AF in unsupervised real-world conditions. The accuracy of AF detection algorithms in this setting is excellent, but PPG-derived heart rate may tend to underestimate higher heart rates.

## Graphical Abstract

**Graphical Abstract:**
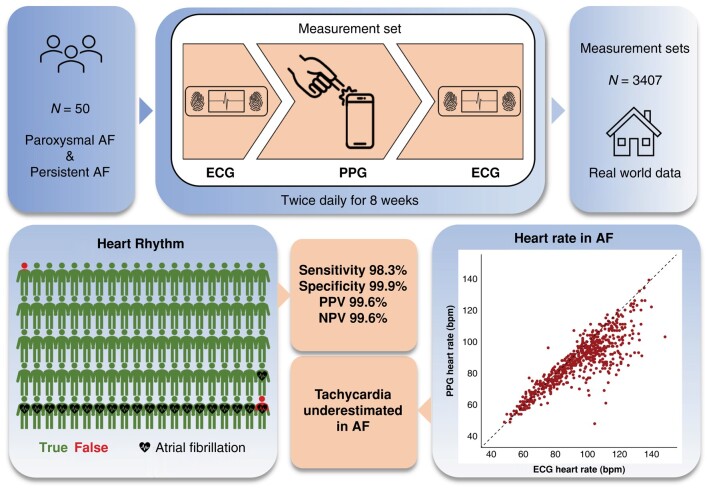

## Introduction

Atrial fibrillation (AF) is the most common arrhythmia with a global prevalence of 2–4%.^1^ It is associated with increased morbidity and mortality, and confers a 5-times increased stroke risk.^2^ The prevalence of AF is expected to continue rising, primarily owing to population aging and the increasing prevalence of AF risk factors, leading to a substantial economic and public health burden.^1^ The diagnosis of AF requires an electrocardiogram (ECG) showing irregularly R–R intervals (when atrioventricular conduction is not impaired) and the absence of distinct repeating *P* waves.^1^ The 2020 European Society of Cardiology (ESC) guidelines for the diagnosis and management of AF proposed an integrated care model, recognizing the use of digital technology and mobile health (mHealth) solutions, to support the diagnosis and management of AF.^1^ Digital devices with technologically advanced mHealth capabilities, are available on the consumer market and have become ubiquitous. The technology used by these devices is mainly based on electrocardiography or photoplethysmography (PPG). Whereas traditionally, heart rhythm measurements were performed with medical ECG devices, these ESC 2020 guidelines have led the way to incorporate the use of single-lead ECG from mHealth devices to diagnose AF. Both the ESC guidelines and a consensus statement by European Heart Rhythm Association (EHRA) in conjunction with the Heart Rhythm Society, recognized the use of PPG-based mHealth devices, combined with artificial intelligence (AI), to have opened up opportunities for the detection and diagnosis of AF and designated it an important frontier in arrhythmia management.^3^ However, the validation of PPG for the diagnosis of AF was identified as one of the gaps in evidence. Data on PPG validation are scarce, and the available studies are affected by high risk of bias.^4^ Moreover, these trials were performed in clinical centres, with measurements performed in a supervised clinical environment.^4–6^ This is very different from the ambulatory real-world environment in which these devices are used. The accuracy has been demonstrated to decrease in unsupervised measurements due to artefacts.^7^ A multitude of research studies have assessed the ability of PPG to screen for asymptomatic AF.^8–10^ However, there is a paucity of data to validate these measurements in ambulatory real-world conditions.^11^ In 2022, the EHRA published a consensus statement that PPG-based devices can be used in screening for AF and monitoring of AF patients.^12^ However, the diagnosis of AF requires confirmation with an ECG after screening or before monitoring with PPG. Reckoning with the EHRA practical guidelines for digital devices, the available data cannot answer the question: ‘How well can a smartphone PPG, analysed by a machine learning algorithm, recognize AF and determine the heart rate, in ambulatory real-world conditions, in patients with an established diagnosis of AF?’

The aim of this study is to validate PPG-derived heart rate and rhythm classification with a smartphone, within the setting endorsed by the EHRA practical guidelines. Therefore, this study focuses on patients with an established diagnosis of AF, performing PPG measurements in unsupervised real-world conditions.

## Methods

### Study design

The RELATION PPG study (REal-worLd vAlidaTION of PhotoPlethysmoGraphy) (ClinicalTrials. gov identifier number NCT06028893) is a pragmatic, prospective, blinded validation study, that was conducted in all patients scheduled for AF ablation at Ziekenhuis Oost-Limburg, (Genk, Belgium) during the inclusion period. The inclusion period was split in period one (27 December 2022 to 7 February 2023) and period two (31 May 2023 to 13 July 2023), due to the availability of handheld ECG devices. Patients aged 18 years or older, without a pacemaker, were screened via telephone contact at least 5 weeks prior to the AF ablation. Patients who met the inclusion criteria (see Supplementary material online, *Table S1*) were invited for an in-person appointment with one of the research team members at the hospital. If patients preferred not to participate, the reason was documented. All patients who presented to the appointment were included. During the first study visit, participants received comprehensive information about the study and provided written informed consent. Participants were assisted to install and configure the ECG and PPG applications on their smartphones and received the ECG hardware. The patients were instructed to perform a ‘measurement set’ twice daily, commencing four weeks prior to the ablation procedure and continuing 4 weeks after the procedure. One measurement set consists of three heart rhythm measurements: an ECG measurement first, followed by a PPG measurement and concluded with a second ECG measurement to assure that the rhythm had not changed (*Graphical Abstract*). The study complies with the Declaration of Helsinki and was approved by the ethical review board (Z-2021058–2) of Ziekenhuis Oost–Limburg (Genk, Belgium).

### Photoplethysmography rhythm measurements

Photoplethysmography measurements were performed using a commercially available application on the patient’s smartphone [Fibricheck (FC), Qompium NV, Hasselt, Belgium] as described elsewhere.^6^ Photoplethysmography is a technique that is well known in clinical practice for its use in fingertip devices to measure oxygen saturation and pulse rate and has now been incorporated in digital devices such as smartphones, smartwatches, and fitness trackers. Photoplethysmography analyses the heart rate and rhythm using an optical technique that measures the peripheral pulse. A light source and a detector are used to measure changes in blood volume within the skin surface, by detecting changes in reflected light intensity, generating a pulse waveform.^13^ Commercially available smartphone applications such as FC can record the PPG waveform using the smartphone camera combined with the LED flashlight for (finger-over-the-camera) PPG. The PPG signal acquisition time on FC was set at 1 min, during which a visible countdown timer was displayed on the smartphone screen. To obtain a PPG measurement, subjects were instructed to initiate a measurement in the FC application and hold the index finger over the flashlight and rear camera without applying excessive pressure. The resulting PPG waveform was analysed by the commercially available FC algorithm (version 1.5.2). The FC algorithm labelled measurements as AF, sinus rhythm (a conglomerate of sinus rhythm, bradycardia, tachycardia, frequent extrasystole, bigeminy, or trigeminy), or insufficient quality. Photoplethysmography traces labelled as insufficient quality were excluded from the analysis. Patients received instant feedback on the quality of PPG measurements, heart rate, and rhythm classification. In case of insufficient quality, the patient was instructed to repeat this process until a measurement of sufficient quality for analysis was recorded.

### ECG rhythm measurements

ECG measurements were performed using a commercially available handheld single-lead ECG device [KardiaMobile (KM) 6L, Alivecor Inc., Mountain View, CA, USA]. Single-lead ECG signals were acquired during 30 s, during which a visible countdown timer was presented in the application. Patients were instructed to hold the KM device in front of them, oriented with the two electrodes facing upwards. ECG recording commenced with finger placement (thumb or index finger and middle finger) of each hand on the respective electrode. After 30 s of signal acquisition, the patient received instant feedback from the KM algorithm on the signal quality and rhythm diagnosis. In case of insufficient quality, the patient was instructed to repeat this process until a measurement of sufficient quality was recorded. The ECG traces were analysed by two blinded cardiologists (H.G. and T.V.P.). In case of uncertainty, a third cardiologist was consulted, blinded for the previous diagnosis (P.H. or L.P.). All cardiologists were blinded for the results of the PPG analysis. Cardiologists labelled the ECG traces as sinus rhythm, AF, atrial flutter/regular atrial tachycardia (AFL/AT), or insufficient quality for analysis. Atrial fibrillation was diagnosed based on the definition described in the ESC 2020 guidelines: ‘Irregularly irregular R-R intervals; Absence of distinct repeating *P* waves; And irregular atrial activations.’^1^ ECG traces labelled as AFL/AT and insufficient quality were excluded from the analysis.

### Analysis

The measurement sets were used to validate the FC algorithm classification (sinus rhythm or AF) against the cardiologist diagnosis on single-lead ECG. If the rhythm changed between the first and second ECG of a measurement set, the measurement set was excluded from the analysis. Sensitivity, specificity, positive predictive values (PPV), and negative predictive values (NPV) were calculated with corresponding 95% confidence intervals using a generalized linear regression model to correct for clustering of data (measurement sets) within patients and compare the performance between groups. The rhythm classification performance was compared in three heart rate zones: bradycardia (<60 b.p.m.), normocardia (60 b.p.m.–100 b.p.m.), and tachycardia (>100 b.p.m.), as well as before and after the AF ablation procedure.

The heart rate assessment with PPG was validated against single-lead ECG, using the average heart rate of the ECG before and after the PPG measurement as reference value. If the heart rate difference between both ECGs was more than ±10% of their average, the measurement set was excluded. We defined an accurate PPG-based heart rate assessment as one that deviated less than ±10% from the corresponding ECG-derived heart rate. The agreement between the PPG-based and ECG-derived heart rate was assessed in sinus rhythm and in AF using the Bland–Altman analysis. Differences were compared using independent-samples *t*-tests. We used the root mean squared error (RMSE) to quantify the disagreement. The RMSE is particularly sensitive to outliers (i.e. clinically relevant mistakes) and is defined as follows: RMSE = $\sqrt{\frac{1}{N}\underset{N}{\sum }\;(\mathrm{PPG}\;\mathrm{heart}\;\mathrm{rate}-\mathrm{ECG}\;\mathrm{heart}\;\mathrm{rate}{)}^{2}}$.^14^ Calculations were performed using IBM SPSS Statistics (version 29, Chicago, IL, USA).

## Results

### Patient population

A total of 91 subjects were screened for eligibility and were contacted (see Supplementary material online, *Figure S1*). Four subjects did not respond, 13 did not possess a smartphone and 22 preferred not to take part in the study due to personal reasons documented in Supplementary material online, *Table S2*. Two subjects did not show up at their inclusion study visits. Fifty subjects were included, patient characteristics are summarized in *Table 1*.

**Table 1:** Baseline and procedural characteristics

Baseline characteristics	*n* = 50
Age (years)	63 (±11)
Sex (male)	34 (68)
BMI (kg/m²)	29 (±5)
CHA_2_DS_2_-VASc score	
0	9 (18)
1	9 (18)
≥2	32 (64)
Congestive heart failure	9 (18)
Arterial hypertension	28 (56)
Diabetes mellitus	10 (20)
Stroke, TIA, or systemic embolism	4 (8)
Venous thromboembolism, pulmonary embolism	2 (4)
Arterial vascular disease	2 (4)
Sleep apnoea syndrome	9 (18)
Coronary artery disease	7 (14)
LVEF	
<40%	2 (4)
40–50%	2 (4)
>50%	46 (92)
Oral anticoagulation	44 (88)
Antiarrhythmic drugs	
Class I	17 (34)
Class II	39 (78)
Class III	18 (36)
Class IV	2 (2)

Procedural characteristics	
Persistent AF	27 (54)
Redo AF ablation	7 (14)
Energy	
Radiofrequency	44 (88)
Pulsed field ablation	6 (12)

Continuous variables are presented as means (±standard deviation). Categorical variables as counts (percentage). The Vaughan Williams classification is used to classify antiarrhythmic drugs.

AF, atrial fibrillation; BMI, body mass index; LVEF, left ventricular ejection fraction; TIA, transient ischaemic attack.

**Figure 1:**
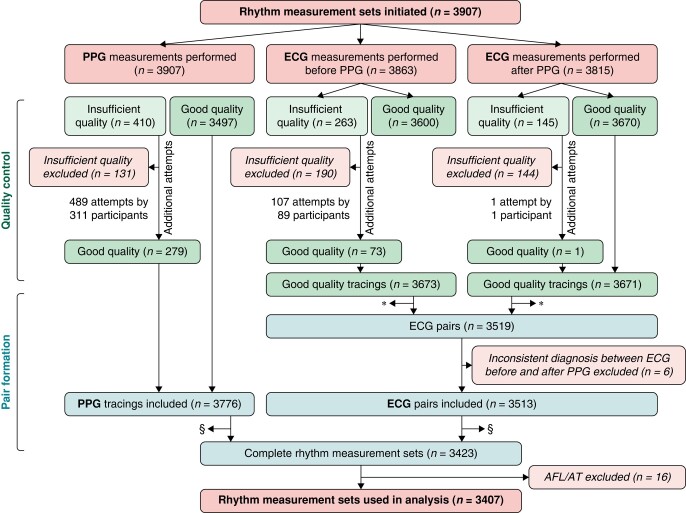
Measurement selection flowchart. ^a^Electrocardiography measurements not forming pairs were excluded; ^b^measurements not forming sets were excluded. AFL, atrial flutter; AT, atrial tachycardia; ECG, electrocardiography; PPG, photoplethysmography.

A total of 3907 measurement sets were performed by all participants, averaging at 78.1 measurement sets per participant or 1.4 measurements sets per day. Because the patients were instructed to perform 2 measurement sets per day, the mean compliance was 69.8% (78.14/112) and median compliance was 82.1% (Q1–Q3, 59.6–92.6%). Measurements were excluded if sufficient quality could not be attained after repeated measurements (PPG, *n* = 131; ECG before PPG, *n* = 190; ECG after PPG, *n* = 144). The resulting measurements were reassembled in 3519 ECG pairs of which six were excluded because the diagnosis between both ECGs was not consistent. Subsequently, the ECG pairs were reassembled with the corresponding PPG measurements forming 3423 complete measurement sets of which 16 were excluded due to AFL/AT. This resulted in 3407 measurement sets used for further analysis (*Figure 1*). Of these, 2684 (78.8%) were in sinus rhythm and 723 (22.2%) were in AF.

### Heart rhythm classification

**Figure 2:**
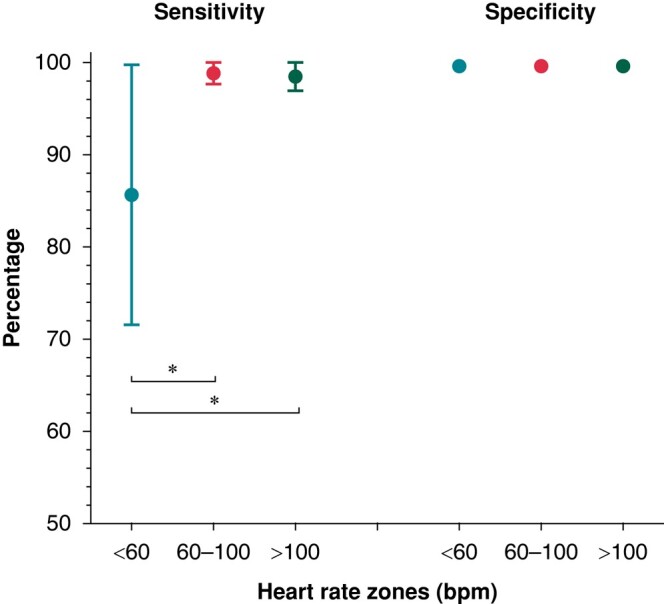
Heart rhythm classification in heart rate zones. Coloured dots represent the sensitivity and specificity for AF detection in the corresponding heart rate zones. Significant differences are indicated with an asterisk (*P-value < 0.001). Vertical lines represent 95% confidence intervals. AF, atrial fibrillation; b.p.m., beats per minute.

The overall sensitivity of PPG to detect AF was 98.3% (95% CI: 96.7–99.9%). The overall specificity of PPG to classify sinus rhythm was 99.9% (95% CI: 99.8–100.0%). The positive predictive value was 99.6% (95% CI: 99.1–100.0%) and the negative predictive value was 99.6% `(99.0–100.0%) for the detection of AF. The contingency table and diagnostic metrics calculated as proportions were reported in Supplementary material online, *Table S3*. There was no significant difference in the rhythm classification performance before or after AF ablation (*P*-value for sensitivity = 0.297; *P*-value specificity = 0.503). There was no significant difference between the specificity of PPG to classify sinus rhythm in all three heart rate zones (*P*-value = 0.178; *Figure 2*). However, the sensitivity to detect AF was significantly lower in bradycardia (<60 b.p.m.; sensitivity 85.2%), compared to normal heart rates (60–100 b.p.m.; sensitivity 99.0%) and tachycardia (>100 b.p.m.; sensitivity 98.7%) (*P*-value < 0.001). The calculated performance metrics were reported in Supplementary material online, *Table S4*.

### Heart rate

**Figure 3:**
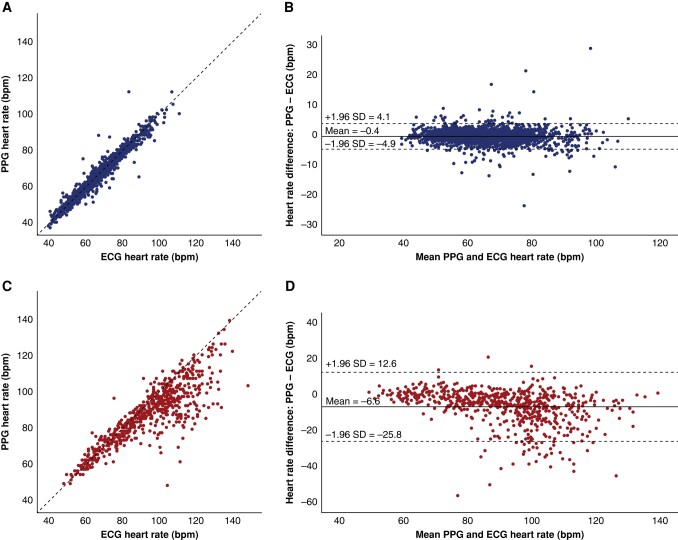
Heart rate assessment. Heart rate agreement between ECG (reference) and PPG. The upper plots (*A* and *B*) are heart rates in sinus rhythm, the lower plots (*C* and *D*) are heart rates in AF. The dashed line in the left upper and lower plots represents a perfect agreement, *y* = *x*. The right upper and lower plots (*C* and *D*) are Bland–Altman plots. The central line represents the mean difference between ECG and PPG, the upper and lower dashed lines represent the Bland–Altman upper and lower limits. b.p.m., beats per minute; ECG, electrocardiography; PPG, photoplethysmography.

The heart rate analysis was performed using all measurement sets with a consistent heart rate between the first and second ECG, following the definition described in the Methods section. Based on these criteria 93.5% of the measurement sets (*n* = 3184/3407) were accepted. The heart rate varied from a minimum of 40 b.p.m. to a maximum of 149 b.p.m. The mean heart rate (71.5 ± 17.3 b.p.m.) was significantly higher during AF (94.7 ± 19.4 b.p.m.) compared to sinus rhythm (65.7 ± 10.7 b.p.m.) (*P*-value < 0.001). The heart rate correlation between PPG and ECG is depicted in *Figure 3*. Photoplethysmography underestimated the heart rate in sinus rhythm with 0.4 b.p.m. (95% CI: −0.3 to −0.5 b.p.m.) and in AF with 6.6 b.p.m. (95% CI: −5.8 to −7.4 b.p.m.). The heart rate difference between PPG and ECG is significantly greater during AF (RMSE = 11.8 b.p.m.) compared to sinus rhythm (RMSE = 2.2 b.p.m.) (*P*-value < 0.001). Heart rate assessment with PPG was accurate within a 10% margin for 2988 (93.8%) measurements out of the 3184 measurement sets included in the heart rate analysis. Inaccurate heart rate assessments were more frequent in AF (161/637; 25.3%) compared to sinus rhythm (35/2547; 1.4%) (*P*-value <0.001). The corresponding Bland–Altman plots (*Figure 3*) demonstrate a skewed underestimation of the heart rate in AF with PPG, particularly for high heart rates.

### Signal quality

The reported performance metrics apply to measurements of sufficient quality for analysis, further referred to as ‘good quality’. The signal quality of single-lead ECG was assessed on the first ECG of the measurement set (conducted prior to the PPG measurement), because the quality of the second ECG of the measurement set could be contaminated with learnings transferred from the first ECG conducted. On the first attempt, ECG was more frequently (93.2%, 3600/3863) of good quality compared to PPG (89.5%, 3497/3907) (*P*-value < 0.001). In response to the ‘insufficient quality’ feedback from the PPG-based smartphone application, 311 participants performed 489 additional attempts to generate a good quality PPG recoding, of these 279 were successful. Likewise, in response to the ‘insufficient quality’ feedback from the single-lead ECG device, 89 participants performed 107 additional attempts to generate a good quality ECG recording, of these 73 were successful. After the repeated measurements, a PPG waveform of good quality was obtained in 3776 (96.6%) out of 3907 PPG measurements. By comparison, an ECG trace of good quality was obtained in 3673 (95.1%) out of 3863 PPG measurements.

## Discussion

**Figure 4:**
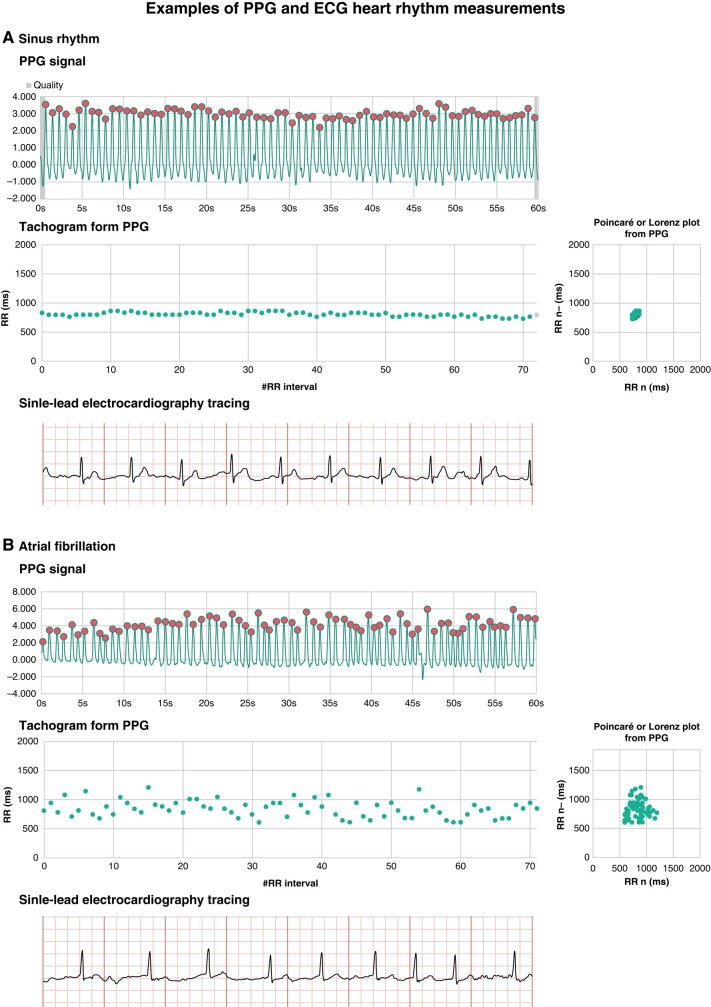
Examples of PPG and ECG heart rhythm measurements. A PPG and single-lead ECG measurement are presented in sinus rhythm (*A*) and AF (*B*). The PPG recordings are presented as raw PPG signals with the tachogram, showing all consecutive pulse signal intervals, and the Poincaré or Lorenz plot, showing the pulse interval as a function of the preceding pulse interval. The single-lead ECG recordings are presented as 8 s tracings at a speed of 25 mm/s and 10 mm/mV. ECG, electrocardiography; PPG, photoplethysmography.

This study validated the performance of a PPG-based smartphone application to differentiate between sinus rhythm and AF in an uncontrolled, real-world setting. Photoplethysmography signals were acquired with the cameras of conventional smartphones using the FC application. The FC application automatically runs a machine learning algorithm that analyses PPG signals. The algorithm performance was excellent, with 98.3% sensitivity, 99.9% specificity, 99.6% PPV, and 99.6% NPV. Sufficient quality PPG could be obtained after a single measurement in 89.5% and increased to 96.6% upon repeated measurements (*Figure 4*). Clinicians utilizing PPG for rhythm analysis should be aware that AF is more frequently missed in bradycardia (i.e. the sensitivity decreases in low heart rates).

This data fills an important evidence gap identified by the ESC 2020 guidelines and is the first documented evidence of PPG performance in the setting for which it is endorsed by the EHRA practical guide for digital devices.^12^ Importantly, this validation was performed in real-world conditions. This type of real-world evidence is important for clinical applicability and is in sheer contrast with the supervised clinical setting of previous validation studies.^4^ The performance of PPG-based rhythm classification decreases in unsupervised recordings in real-world conditions.^7^ Only one (abstract) publication evaluated smartphone PPG-based AF detection in a real-world setting.^11^ The setting in which measurements were performed was the major issue with contemporary validation studies along with a high risk of selection bias in 65% of the studies. In the remaining 35%, selection bias could not be assessed. Moreover, there was a lack of clarity in the methodology of these studies, particularly in terms of data exclusion criteria and the timing of reference and index tests.^4^ In summary, no previous study met all Quality Assessment of Diagnostic Accuracy Studies (QUADAS)-2 criteria.^15^

This validation study meets all QUADAS-2 criteria and allows clinicians and researchers to gauge the accuracy of PPG-based rhythm classification. However, the results of this study are valid for the FC application and algorithm only. There are several other smartphone applications for AF detection (Cardiio Rhythm, Preventicus, PULSE-SMART, Happitech) but PPG signal acquirement might differ between applications and the rhythm classification algorithms are AI ‘black boxes’ in all applications. Moreover, algorithm updates are likely to change the performance continuously. Hence, the need for individual validation studies.^4,16,17^ Currently, FC is the only smartphone application cleared by the Food and Drug Administration and FC, Preventicus and Happitech have Conformité Européenne (CE) approval. A head-to-head comparison study between FC and Preventicus is currently being conducted (VALIDATION, NCT06023290). Besides PPG algorithms, the FC application provides a waveform and plots that allow visual interpretation by physicians (*Figure 4*).^18,19^ A distinction should be made between PPG applications for smartphones and wrist worn devices. The latter have the obvious advantage of more continuous monitoring and disadvantage of increased background noise.^7^ Hence, the applications require other algorithms, separate validations and are more suitable for different populations. In most populations, AF prevalence will be lower than 22.2%, as found in this study, decreasing the PPV and increasing the NPV.^18^ Defining which device is best for which setting requires further study that should inevitably start with an unbiased validation in the target population and setting. The populations not included in this study were patients with a pacemaker and patients who do not possess a personal smartphone. For these patients, other AF monitoring techniques might be more appropriate. Photoplethysmography-based applications have mainly been used in three settings.

One, PPG-based applications have previously been used in the setting of AF screening, targeting large populations. However, to date, PPG has the disadvantage of requiring ECG to confirm the diagnosis. Confirming the diagnosis appeared to be challenging in these studies because an ECG required additional hardware and testing that not all patients were willing to do. Moreover, the time lag between PPG and ECG might permit the AF episode to terminate. This led to a wide variety of AF confirmation rates ranging from 34% in the Apple Heart Study, 36% in the eBRAVE study, 60% in the ‘Smart in OAC—AFNET 9’ study to 87% in the Huawei Heart Study.^8–10,20–22^

Two, PPG-based applications are useful not only for screening but also for tracking episodes among those with an established diagnosis of AF. In that setting, major life-changing decisions, such as whether to initiate an anticoagulant, do not hinge on the results from PPG measurements. Instead, real-time AF detections can empower patients to identify triggers of their AF, to assess the effectiveness therapy, and to determine whether symptoms are attributable to arrhythmia or something else.^23^ This was first demonstrated by the TeleCheck-AF project during the COVID-19 pandemic, when patients were unable to visit a physician.^24^ After the COVID-19 pandemic, PPG remained an attractive solution to monitor AF recurrence after ablation;^25,26^and to monitor AF burden with intermittent or continuous PPG.^27^

Three, PPG-based applications could potentially be of use to guide rate control in subjects with persistent or permanent AF. The accuracy of PPG-derived heart rate in AF has been studied in real-world conditions with wearable PPG devices, but not with smartphones. A recent study performed with smartwatches reported an accurate PPG-derived heart rate with 95% of measurements within −8.4 b.p.m. to +9.9 b.p.m. compared to the ground truth (Bland–Altman upper and lower boundaries). The RMSE in this study was 4.4 b.p.m.^14^ In our study, PPG-derived heart rate was less accurate with a RMSE of 11.8 b.p.m. and Bland–Altman boundaries at −25.8 to +12 b.p.m. In a smartwatch study, the majority of PPG measurements are acquired at night during sleep, when there are less movement artefacts, and the heart rate is at its lowest. Because the mean heart rate in this study (75 b.p.m.) was lower compared to our study (95 b.p.m.) and the inaccuracy of PPG-derived heart rate increases at higher rates, PPG-derived heart rate was less accurate in our study.

Why PPG tends to underestimate high heart rates in the presence of AF, can be elucidated from the haemodynamic mechanisms fundamental to PPG. Photoplethysmography waveform construction relies on variations in skin colour caused by the propagation of blood with every heartbeat. In short RR intervals, compromised ventricular filling may lead to a reduction in stroke volume, resulting in a change in skin colour that falls below the detection limit.^28^ Because PPG wave detection calibrates the detection limit to the previous waves, this is more likely to occur in irregular rhythms with a mixture of both large and small PPG waves. Whether this limitation is inherent to PPG technology, or to the PPG algorithms employed in PPG devices, requires further investigation.

The future of AF management is likely to hold a more integrated approach, as recommended in the ESC 2020 guidelines.^1^ Digitalization of AF management programs is appealing to make such integrated approach feasible.^29^ While research on digitalization is experiencing rapid and extensive growth,^30^ further studies are needed to inform clinicians on the performance and limitations of the digital tools and to clarify whether this will lead to better outcomes.

### Limitations

This study is best understood within the confinements of its limitations. First are the limitations inherent to smartphone-based PPG. This modality requires a smartphone. Since patients used their personal smartphone in this study, these results only apply to patients possessing and capable to operate a smartphone. Moreover, this validation applies only to the discrimination between sinus rhythm and AF by the FC algorithm (version 1.5.2), in good quality PPG signals. Hence, a significant amount of data was excluded due to poor quality. There are factors that can influence signal-to-noise ratio, such as tremor. However, this obstacle is circumnavigated by the automated signal quality check in the FC application. In addition, this study has demonstrated that sufficient signal quality can almost always be obtained upon repeated measurements. Other arrhythmias such as AT/AFL were not studied as most digital application does not provide a specific classification for AT/AFL. Currently, AT/AFL is classified by FC and KM under the umbrella term ‘tachycardia’, attempts for specific labelling are not validated. This should be an area of focus and future algorithm development. Although the diagnosis of AF was based on objective criteria as stated in the methods, a limitation can be seen in the fact that ECG labelling by cardiologists is by definition prone to subjective interpretation to some degree and the fact that PPG signals and ECG recordings were not performed simultaneously, but successively. Although a change of heart rate during the measurement set seemed unlikely, this limitation was addressed by performing a second ECG and excluding the rare cases with a change in heart rate or rhythm. As opposed to a change rhythm, a change in heart rate during the measurement set is more likely, therefore heart rate variations during the measurement sets were evaluated and excluded as described in the Methods section.

The study was not designed to evaluated patient compliance to PPG monitoring because patients were requested to perform PPG measurements in combination with two ECG measurements, which is more laborious and might negatively affect patient compliance. Despite, the compliance was comparable to other studies using PPG and reporting a compliance of 60.5% (mean over 7 days) and 69.7% (median over 6 months).^10,22^

## Conclusion

In conclusion, the detection of AF using smartphone-derived PPG signals proves to be highly accurate. Clinicians can place confidence in the rhythm classification distinguishing between sinus rhythm and AF, as well as in the PPG-derived heart rate measurements performed by digitally literate AF ablation patients in an unsupervised real-world setting. However, PPG measurements may underestimate high heart rates in the presence of AF.

## Supplementary Material

euae065_Supplementary_Data

## Data Availability

The data that support the findings of this study are available from the corresponding author, upon reasonable request.
