# Supplementary material for: Real-world validation of smartphone-based photoplethysmography for rate and rhythm monitoring in atrial fibrillation
Source: Europace. 2024 Apr 17;26(4):euae065. doi: 10.1093/europace/euae065 (PMC11023210; doi:10.1093/europace/euae065)
Supplement: euae065_Supplementary_Data [file euae065_supplementary_data.pdf]

Supplementary Tables and Figures

Supplementary Table S1 : Inclusion and exclusion criteria 1

Supplementary figure S1 : Patient screening and inclusion flowchart 1

Supplementary Table S2 : Screening Failures due to Personal Reasons 2

Supplementary Table S3 : Contingency table and diagnostic metrics of the PPG algorithm calculated as proportions 2

Supplementary Table S4 : Diagnostic metrics of the PPG algorithm by group.....................3

Supplementary Table S1 : Inclusion and exclusion criteria

Inclusion criteria	Exclusion criteria
Subject is at least 18 years of age.	Insufficient cognitive or comprehensive level of Dutch to participate to the trial.
Subject understands, agrees with, and signs the informed consent.	No access to a smartphone at home.
Subject is scheduled for an ablation procedure for AF during the inclusion period.	Subject has a pacemaker.

Supplementary Figure S1 : Patient screening and inclusion flowchart

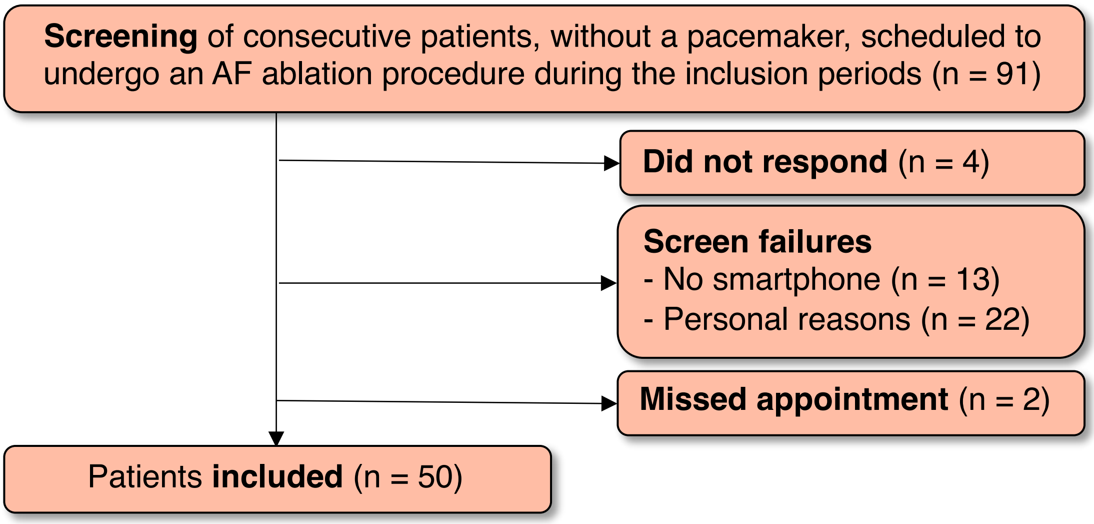

Supplementary Table S2 : Screening Failures due to Personal Reasons

Screening Failures due to Personal Reasons (n = 22)	
**Reason for Screen Failure**	**Amount of Screen Failures (n)**
Not interested	8
Vacation	4
Uses other monitoring method	3
Language barrier	2
No time	2
Poor technical knowledge	2
Anxiety due to measurements	1

Supplementary Table S3 : Contingency table and diagnostic metrics of the PPG algorithm calculated as proportions

	ECG – AF	ECG – SR	Total	
PPG – AF	711	3	714	**PPV** = 711 / 714 **= 99.6%**
PPG – SR	12	2681	2693	**NPV** = 2681 / 2693 **= 99.6%**
Total	723	2684	3407	
	**Sensitivity**<br>= 711 / 723<br>**= 98.3%**	**Specificity**<br>= 2681 / 2684<br>**= 99.9%**		

AF, atrial fibrillation; ECG, electrocardiography; NPV, negative predictive value; PPG, photoplethysmography, PPV, positive predictive value; SR, sinus rhythm

Supplementary Table S4 : Diagnostic metrics of the PPG algorithm by group

	Sensitivity	95% CI LL	95% CI UL	Specificity	95%CI LL	95% CI UL
Before AF ablation	0.9899	0.9734	1.000	0.9984	0.9961	1.000
After AF ablation	0.9693	0.9247	1.000	0.9993	0.9979	1.000
Heart rate zone 1	0.8519	0.7179	0.9857	0.9976	0.9942	1.000
Heart rate zone 2	0.9897	0.9773	1.000	0.9994	0.9984	1.000
Heart rate zone 3	0.9869	0.9688	1.000	1.000	/	/

	p-value for sensitivity	p-value for specificity
Before vs after AF ablation	0.2966	0.5031
Heart rate zone 1 vs 2	< 0.0001	0.1783
Heart rate zone 2 vs 3	0.7050	/
Heart rate zone 1 vs 3	< 0.0001	/

Heart Rate zones are <60bpm, 60bpm-100bpm and >100bpm for zone 1,2 and 3 respectively. AF, atrial fibrillation; CI, confidence interval; LL, lower limit; UL, upper limit.
